# A New LC-MS/MS-Based Method for the Simultaneous Detection of α-Tocopherol and Its Long-Chain Metabolites in Plasma Samples Using Stable Isotope Dilution Analysis

**DOI:** 10.3390/ph17111405

**Published:** 2024-10-22

**Authors:** Alexander Maxones, Eva Beck, Gerald Rimbach, Marc Birringer

**Affiliations:** 1 Department of Nutritional, Food and Consumer Sciences, Fulda University of Applied Sciences, 36037 Fulda, Germany; alexander.maxones@oe.hs-fulda.de (A.M.); eva.m.beck@fau.de (E.B.); 2 Institute of Human Nutrition and Food Science, University of Kiel, 24118 Kiel, Germany; rimbach@foodsci.uni-kiel.de; 3 Food Chemistry, Department of Chemistry and Pharmacy, Friedrich-Alexander-Universität Erlangen-Nürnberg (FAU), 91058 Erlangen, Germany; 4 Public Health Zentrum Fulda (PHZF), Fulda University of Applied Sciences, 36037 Fulda, Germany; 5 Wissenschaftliches Zentrum für Ernährung, Lebensmittel und Nachhaltige Versorgungssysteme (ELVe), Fulda University of Applied Sciences, 36037 Fulda, Germany

**Keywords:** vitamin E, long chain metabolites, liquid-chromatography tandem-mass spectrometry (LC-MS/MS), stable isotope dilution analysis

## Abstract

**Background:** Our study presented a novel LC-MS/MS method for the simultaneous quantification of α-tocopherol (α-TOH) and its phase II metabolites, α-13′-COOH and α-13′-OH, in human serum using deuterium-labeled internal standards (d_6_-α-TOH, d_6_-α-13′-COOH, d_6_-α-13′-OH). **Methods:** The method addresses the analytical challenge posed by the significantly different concentration ranges of α-TOH (µmol/L) and its metabolites (nmol/L). Previous methods quantified these analytes separately, which caused an increase in workflow complexity. **Results:** Key features include the synthesis of stable isotope-labeled standards and the use of a pentafluorophenyl-based core-shell chromatography column for baseline separation of both α-TOH and its metabolites. Additionally, solid phase extraction (SPE) with a HybridSPE^®^ material provides a streamlined sample preparation, enhancing analyte recovery and improving sensitivity. By utilizing deuterium-labeled standards, the method compensates for matrix effects and ion suppression. This new approach achieves precise and accurate measurements with limits of detection (LOD) and quantification (LOQ), similar to previous studies. Calibration, accuracy, and precision parameters align well with the existing literature. **Conclusions:** Our method offers significant advantages in the simultaneous analysis of tocopherol and its metabolites despite concentration differences spanning up to three orders of magnitude. In contrast to earlier studies, which required separate sample preparations and analytical techniques for tocopherol and its metabolites, our approach streamlines this process. The use of a solid-phase extraction procedure allows for parallel sample preparation. This not only enhances efficiency but also significantly accelerates pre-analytical workflows, making the method highly suitable for large-scale studies.

## 1. Introduction

α-Tocopherol and other vitamin E vitamers containing a chromanol ring structure with a saturated or unsaturated side chain are lipid-soluble antioxidants that prevent the oxidation of unsaturated fatty acids by reactive oxygen species (ROS) [1].

In the last decade, research on vitamin E metabolism fostered the understanding of molecular structures and biological functions of its metabolites. Besides the urinary metabolite α-carboxy-ethyl-hydroxychromanol (α-CEHC) that has been suggested as a marker of an adequate vitamin E uptake [2], long-chain metabolites (LCMs) of α-tocopherol, 13′-hydroxy- and 13′-carboxychromanol (α-13′-OH and α-13′-COOH), have been detected in human feces and human plasma at nanomolar concentrations [3,4,5,6,7,8]. LCMs have shown anti-inflammatory, anti-proliferative, and lipid regulatory properties [3,5,9]. Furthermore, long-chain metabolites were found to induce accelerated wound healing and diminish inflammation in a mouse model of atopic dermatitis [10,11]. As a possible target in the inflammatory network, Pein et al. found the α-tocopherol metabolite α-13′-COOH a highly effective endogenous inhibitor of 5-lipoxygenase (5-LO) [12]. The concentration of LCMs seems dependent on the circulating amount of tocopherol in the plasma. A supplementation of 800 IU/d *RRR*-α-tocopherol for 1 week resulted in a 10–15-fold increase in LCMs [8].

Vitamin E metabolite research has been accelerated by the ease of metabolite synthesis based on the isolation of garcinoic acid from the African bitter nut *Garcinia cola*. A simple, three-step synthesis starting from garcinoic acid (δ-13′-COOH-tocotrienolic acid) resulted in reasonable yields of α-13′-hydroxychromanol (α-13′-OH) and α-13′-carboxychromanol (α-13′-COOH), respectively. As a result, partially deuterated metabolites were synthesized to serve as internal isotope labeled standards (ILSs) in LC-MS-based isotope dilution analyses [6]. Today, most of the recent studies have used UHPLC-MS/MS or -HRMS-based detection methods for the determination of serum metabolites. Zhao et al. used LC-ESI-MS analyses for the detection of vitamin E metabolites in mouse and human urine and plasma, respectively. In the past, pre-analytics consisted of enzymatic degradation of phase II conjugates by glucuronidase and sulfatase followed by a liquid–liquid extraction either by ethyl acetate or a mixture of hexane/tert-butylmethylether. Since the serum and plasma concentration of α-tocopherol and its metabolites vary between two to three orders of magnitude (µM vs. nM), chromatographic conditions and MS-detection setups were conducted in laborious separate workflows [6,7,8]. Here, we present a stable isotope dilution method using deuterated α-tocopherol and deuterated long-chain metabolites for the simultaneous detection and quantification via LC-MS/MS. A new solid-phase extraction procedure was applied to reduce background signals and improve sensitivity. Using a pentafluorophenyl(PF-5)-based core-shell chromatographic column, we were able to separate and quantify LCMs 13′-COOH, 13′-OH and tocopherol within the same run.

## 2. Results

### 2.1. Synthesis

**Figure 1:**
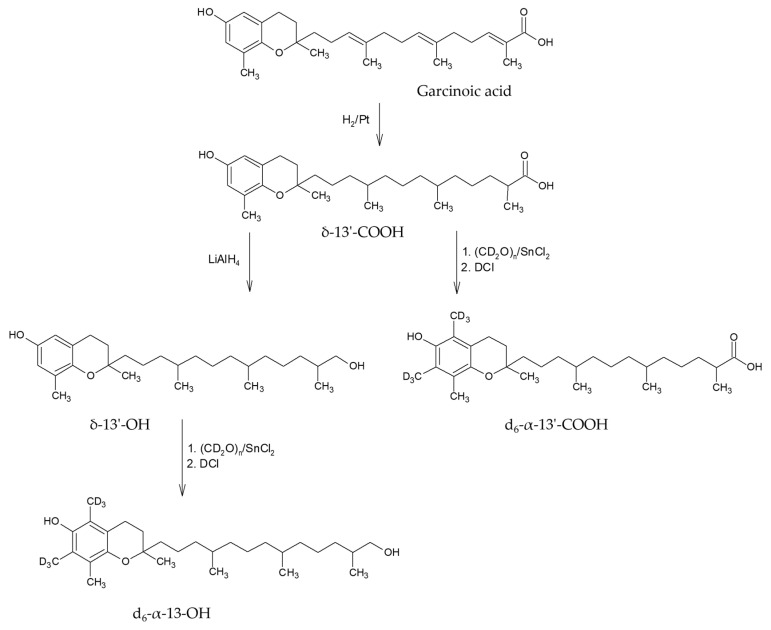
Synthesis of stable isotope labeled long-chain metabolites as an internal standard for liquid chromatography-mass spectrometry.

The synthesis of d_6_-α-tocopherol and deuterated long-chain metabolites is based on the deuteromethylation of δ-tocopherol and its metabolites (Figure 1) [6]. The yield of purified d_6_-α-tocopherol was 36.2%, with 99.0% purity (determined by HPLC-MS). Deuteromethylation of δ-13′-COOH and δ-13′-OH yielded 48.4% and 8.1% of d_6_-α-13′-COOH (95.9% purity) and d_6_-α-13′-OH (99.8% purity), respectively.

### 2.2. Work Up Procedure for Human Serum and Chromatographic Separation of Analytes

Compared with previous studies, we describe several changes in the preparation of the sample matrix, the extraction method, and the measurement procedures of the analytes [6,7,8]. To our knowledge, this is the first time that solid-phase extraction has been described as an alternative to liquid–liquid extraction. In general, solid-phase extraction enables a parallel workflow, in contrast to the sequential process required for liquid–liquid extraction. Besides a significant reduction of the work-up time, the elimination of phospholipids, due to their interaction with the HybridSPE^®^ silica core material, results in an improved background signal.

The use of a core-shell (PF-5) material for chromatographic separation allowed us to determine the long-chain metabolites and the tocopherols within the same chromatographic run. All metabolites (except of the labelled analogues) were base line separated and a complete run took 20 min.

### 2.3. Suitability of Stable Isotope Labeled Standards (ILSs)

**Figure 2:**
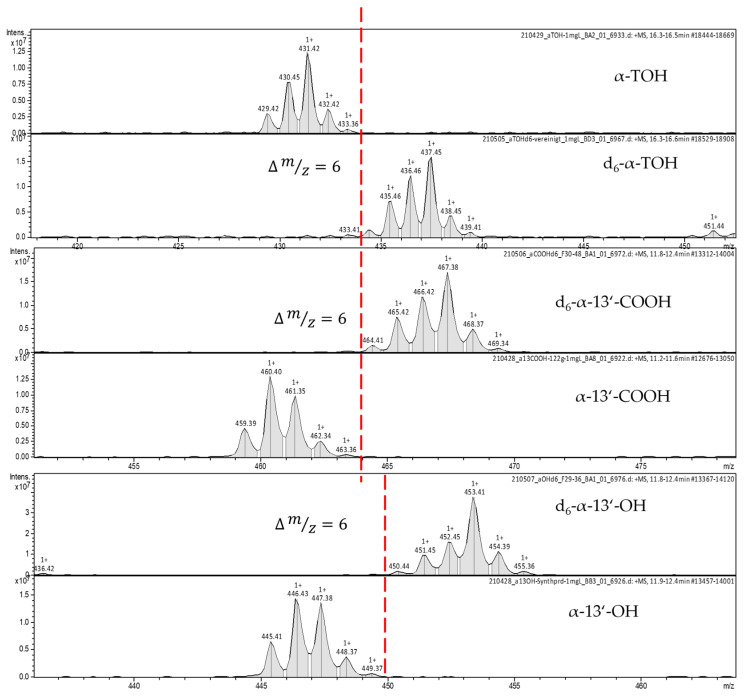
Isotope pattern [M+H]^+^ of labeled and unlabeled tocopherol metabolites.

**Figure 3:**
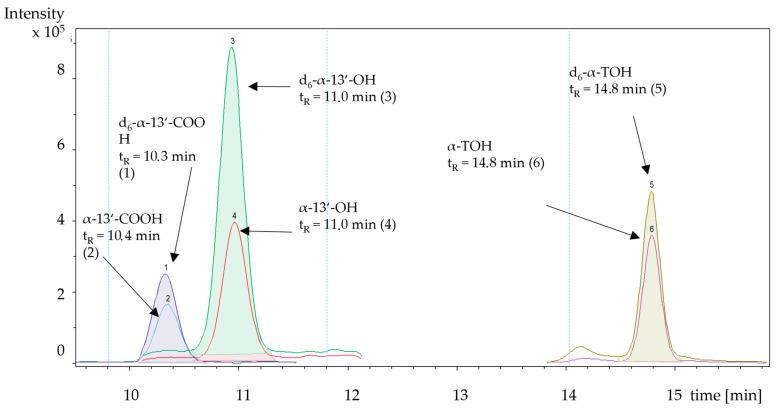
Chromatographic separation of α-tocopherol and its metabolites. Labeled internal standards showed identical retention times. t_R_ = retention time.

In earlier studies, d_4_-labeled standards have been used as ILS with the potential of isobaric mass overlapping [6,7,8]. The mass spectra [M+H]^+^ of the synthesized d_6_-labeled standards show no spectral overlapping (Figure 2) with unlabeled metabolites. However, the retention time remained the same (Figure 3). The retention time of d_6_-α-13′-COOH and α-13′-COOH was 10.4 min, that of d_6_-α-13′-OH and α-13′-OH was 11.0 min, and that d_6_-α-TOH and α-TOH was 14.8 min (Table 1). To circumvent the non-linear response of the analyte in the presence of ILSs, we added 100 pmol of d_6_-metabolites and 2.29 nmol d_6_-α-tocopherol [13]. During the time the study was carried out, we could not detect any D/H-exchange reactions within the labeled standards. The synthesized standards were tested for the absence of the corresponding non-labeled compounds. For the long-chain metabolites, levels were found to be below 0.5%, while for labeled α-tocopherol, the non-labeled counterpart was present at levels below 1% (see Table S1 in Supplementary Material). These levels were deemed suitable for use as internal standards, as they are negligible during sample preparation and use, given the dilution effect during the analytical process.

### 2.4. Method Validation

Table 1 summarizes the method characteristics. LOD and LOQ were calculated at 5.3 and 17.8 nmol/L for α-13′-COOH and 3.4 and 11.2 nmol/L for α-13′-OH, respectively. Compared with earlier studies, the detection and quantification limits for these metabolites were improved by a factor of 15–20 [6]. The limits for α-TOH were calculated as 3.5 and 11.6 µmol/L. The precision (within-run, RSDr) for all deuterated metabolites is less than 15% and thus within the ranges required by the Food and Drug Administration guidelines (FDA) [14]. In addition, the accuracy of our measurements was in the range between 88% and 94%. Calibration curves were generated both in solvent and in the matrix. Response linearity was determined using equidistant concentrations of standards. All response curves showed a coefficient of determination (*R*^2^) higher than 0.99.

### 2.5. MS^n^ for Simultaneous Metabolite and Tocopherol Quantification

**Figure 4:**
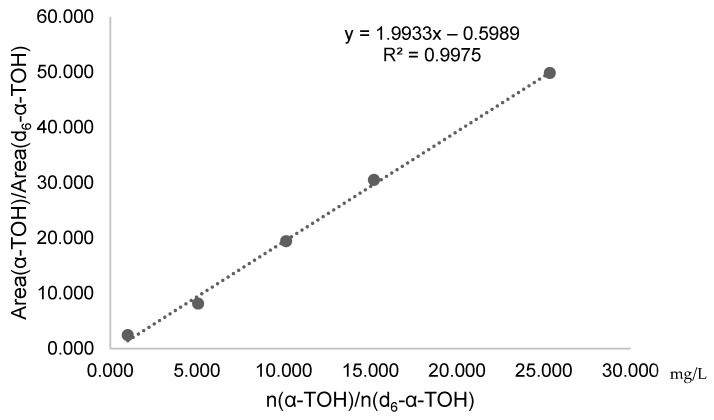
Calibration curve for α-TOH quantification. Areas correspond to MS^3^ transitions (137.0 for α-TOH and 143.0 for d_6_-α-TOH). Coefficient of determination, R^2^.

For the identification and quantification of the labeled and unlabeled metabolites, corresponding qualifiers and quantifiers are indicated in Table 1. As mentioned above, a mass shift of *m*/*z* of 6 leads to a separation of the qualifier masses with no isobaric interferences (i.e., α-13′-COOH [M+H]^+^ 461.3 and d_6_-α-13′-COOH [M+H]^+^ 467.3). To determine the α-tocopherol concentration in the same chromatographic run, we used MS^3^ transitions (431.30 → 165.00 → 137.00 for α-TOH and 437.30 → 171.0 → 143 for d_6_-α-TOH, respectively) to “dilute” the signal and obtain a linear response at the detector (Table 1). A calibration curve (Figure 4) shows the linear response of the transition states (ratio of areas) to the analytes concentrations (ratio). The concentrations of tocopherols are between 1 and 30 mg/L and, thus, 3 magnitudes higher than the expected metabolite concentrations.

## 3. Discussion

The present work describes a new method for the simultaneous analysis of α-tocopherol and its metabolites, namely α-13′COOH and α-13′-OH using stable isotope-labeled internal standards. The focus is on the synthesis, characterization and suitability of stable isotope-labeled standards. Previous studies on the analysis of vitamin E metabolites using LC-MS-based methods determine tocopherol and the metabolites in separate workflows. To the best of our knowledge, this is the first time that we have demonstrated a method to quantify the metabolites in the trace range (nmol/L) and simultaneously α-TOH in the µmol/L range.

The simple synthesis and availability of deuterated α-tocopherol and its metabolites, d_6_-α-13′-COOH and d_6_-α-13′-OH, is a crucial step to ensure accurate quantification. The use of garcinoic acid as a precursor for the synthesis of δ-13′-COOH and δ-13′-OH metabolites and the use of δ-TOH as a precursor of deuterated α-TOH represents a straightforward approach to generate these standards. However, the different yields of the synthesized compounds underline the need for optimization of the synthesis procedures.

Another feature of the method presented here is the sample preparation by solid phase extraction (SPE) instead of conventional liquid–liquid extraction methods. After enzymatic digestion of the sample using sulphatase and β-glucuronidase, phase II-conjugated metabolites are cleaved, released and added directly to a HybridSPE^®^ column. The use of HybridSPE^®^ offers two major advantages over liquid–liquid extraction. Firstly, proteins can be precipitated directly onto the solid phase column (not applied in this study) and through the interaction with the zirconium-coated silica material, phospholipids are retained, resulting in an improved background signal. The workload is optimized, and the samples can be concentrated before analysis. The use of a pentafluorophenyl-based core-shell chromatography column has proven successful for the separation of tocopherols, and we were able to show that the metabolites could also be baseline-separated and measured at acceptable times [15].

The deuterated standards show almost identical retention times to the analytes. This property characterizes a good ILS. In addition, there should be no isobaric overlaps in the mass spectra of analytes and ILS. Due to a mass difference of ∆ *m*/*z* = 6, this could be guaranteed by our labeled standards. It has been shown that isotopically labeled standards are particularly well suited for UHPLC-MS/MS measurements when high-resolution mass spectrometers are not available [16].

The concentration of α-TOH in human plasma typically ranges between 12 and 30 µmol/L and, thus, is about three magnitudes higher than that of the metabolites, which are in the nmol/L range. This fact leads to an analytical dilemma, as the concentration of the SPE can enrich the metabolites of the tocopherols but at the same time lead to a concentration of the tocopherol. This causes a saturation of the α-TOH signal at the detector, which can result in an underestimation of the α-TOH concentration. For this reason, α-TOH and its metabolites were quantified separately in the past, which prolonged the workflow [6,7,8].

Quantification by MS^2^ or MS^3^ transition signals also failed since the signals are subject to strong fluctuations due to matrix effects and ion suppression [17,18,19]. In this study, we were able to show that by using d_6_-α-TOH, fluctuations due to matrix effects or ion suppression in the MS^3^ transitions could be almost eliminated [13]. This study demonstrates the ability of the LC-MS/MS system to accurately differentiate and quantify these compounds within a single chromatographic run, which is critical for comprehensive metabolic profiling and could significantly reduce the workload for studies with larger numbers of subjects.

The introduction of a stable isotope dilution method using these deuterated standards in LC-MS/MS analyses represents a sophisticated strategy to achieve high accuracy and precision in the quantification of α-tocopherol and its metabolites in human serum. The calibration curves, recovery rates, limits of detection (LOD) and limits of quantification (LOQ) determined with this method correspond to those found in the literature [6,7,8]. Table 2 summarizes the concentration values of the metabolites from human serum and the validation parameters from previous studies. In the context of measurement accuracy, we found similar values for α-13′-COOH and α-TOH but not for α-13′-OH. The values determined for the hydroxy-metabolite are 5–7 higher than those found by Giusepponi et al. [6]. One explanation for this difference is the carrier effect of stable isotope standards, which allow a higher recovery of analytes [19]. In contrast to the study by Giusepponi et al., we were able to use a deuterium-labeled α-13′-OH standard, which explains the carrier effect and, thus, the higher amounts of the analyte.

Several studies report isomeric forms of α-13′-COOH (namely M1, M2) and α-13′-OH (M3), which differ only by a shift in retention time but not in the mass spectra [6,7,8]. Interestingly, M1 exceeds the amount of 13′-COOH by 10-fold. The nature of the metabolites M1-M3 is unknown so far and needs to be structurally elucidated in the future. Speculating on the nature of these metabolites leads to structural isomers in which the functional groups -COOH or -OH are not located at position 13′ but within the side chain. As an example, hydroxylated metabolites (12′-OH and 11′-OH) were found in the feces of mice [20], and sargachromenols, a class of meroditerpenes from brown algae, are carboxylated at position 15′ [21]. These isomeric compounds would give the same mass spectra but differ in retention times. In the present study, we could not find these isomeric forms of the metabolites. We assume that the hypothetical structural isomers that could be separated by the C18 column used in the studies by the group of Galli et al. are not separated on our PF-5 Phase. In the direct study comparison, the plasma values of the metabolites are given as sum parameters (α-13′-COOH + M1 + M2 or α-13′-OH + M3) (see Table 2). We found a reduced amount of α-13′-COOH for the endogenous and the supplemented plasma samples. Only 25% of α-13′-COOH from the endogenous plasma and 67% of α-13′-COOH from supplemented plasma could be found. A reduced recovery rate or prolonged storage time (2017 vs. 2021 at −80 °C) could be a reason for the reduced values. However, our data are in good agreement with the plasma α-TOH values from the study by Giusepponi et al. [6].

## 4. Materials and Methods

### 4.1. Chemicals and Reagents

All chemicals were used as received from the supplier. Lyophilized β-glucuronidase (*Escherichia coli* Typ IX-A, 1–5 Mio. units/g), sulfatase (*Helix Pomatia* Typ H-1, ≥10.000 units/g), SnCl2, DCl (35 wt.% in D_2_O, ≥99 Atom% D), paraformaldehyde-d_2_ (98 Atom% D), (+)-δ-Tocopherol (~90%), formic acid (FA) (MS grade), and HybridSPE-Phospholipid Ultra Cartridges (bed wt. 30 mg, volume 1 mL) were received from Sigma Aldrich, Taufkirchen, Germany. LC-MS-grade water and acetonitrile (both LiChrosolv), ascorbic acid (AA), and *all-rac*-α-tocopherol (95%) were obtained from Merck KGaA, Darmstadt, Germany, methanol (HiPerSolv) was obtained from VWR Chemicals, Darmstadt, Germany, and deuteriumoxide (≥99.8 Atom% D) was obtained from Carl Roth, Karlsruhe, Germany. Isolation and purification of garcinoic acid (GA) and synthesis of δ-13′-COOH and δ-13′-OH were performed as described earlier [22,23].

### 4.2. Synthesis and Purification of Deuterated Standards

Deuteromethylation of δ-tocopherol, δ-13′-COOH and δ-13′-OH was done according to known literature procedures [23,24]. In brief, δ-tocopherol was treated with deutero-paraformaldehyde (CD_2_O)_n_ and SnCl_2_ and quenched with DCl/D_2_O to yield d_6_-α-TOH. After purification on silica gel with hexane/ethyl acetate (5:1 *v*/*v*), we obtained d_6_-α-TOH in 36% yield and 99.0% purity. D_6_-α-13′-COOH (d_6_-α-13′-carboxychromanol,(13-((2R)-6-hydroxy-2,5,7,8-tetramethylchroman-2-yl)-2,6,10-tri-methyltridecanoic acid) and d_6_-α-13′-OH (d_6_-α-13′-hydroxychromanol, (13-((2R)-6-hydroxy-2,5,7,8-tetramethylchroman-2-yl)-2,6,10-trimethyltridecanol) were prepared in the same manner from δ-13′-COOH and δ-13′-OH, respectively. After purification on silica gel with hexane/ethyl acetate (3:1 *v*/*v*), we obtained d_6_-α-13′-COOH in 48% yield and 95.9% purity and d_6_-α-13′-OH in 8% yield and 99.8% purity. The synthesized standards were assessed for their purity using LC-MS, with particular focus on the purity concerning foreign substances, such as the used starting materials and purification impurities, as well as the absence of the corresponding non-labeled compounds (see Figures S1–S3 in Supplementary Material). This ensured that the labeled standard substances were present exclusively in the desired isotopic composition.

### 4.3. Quantification of Stable Isotope Labeled Standard Stock Solutions

Calibration curves of unlabeled standards of α-TOH, α-13′-COOH, and α-13′-OH were used to determine the concentration of ILS stock solutions. All quantifications were performed with HPLC-FD at λ_Ex_ = 296 nm and λ_Em_ = 325 nm. The concentration of the stock solutions of d_6_-α-TOH, d_6_-α-13′-COOH, and d_6_-α-13′-OH were 36.42 mg/L, 57.83 mg/L, and 54.07 mg/L, respectively. Aliquots at 1 mg/L in 70% methanol (MeOH/H_2_O 70/30 (*v*/*v*), 1 mg/mL AA and 0.1% FA) were stored under argon at −20 °C.

### 4.4. Plasma Sample Preparation and Isotope Dilution Method

Blood sampling and supplementation have been described in detail [6]. In fact, we used the exact same blood samples of subject 1 in the study of Giuseppino et al. In brief, the volunteer was supplemented with 1000 IU RRR-α-TOH per day for one week using a soft gel capsule formulation of 671 mg RRR-α-TOH in soy oil and sorbitol (Optovits, Hermes Arzneimittel, Germany). Blood sampling was carried out on fasted volunteers. Samples were prepared using serum Monovettes (Sarstedt, Nümbrecht, Germany) by centrifugation (2000× *g*) of blood for 10 min at 20 °C. Samples were then maintained at −80 °C until analysis. The study was approved by the local Ethics committee, “Comitato Universitario di Bioetica dell’Università degli Studi di Perugia” (prot.N.2015-008).

A total of 700 µL of serum was used to determine α-TOH and its long-chain metabolite concentration in triplicate. The sample was treated with 42 µL of ascorbic acid (250 mg/mL) and spiked with 175 µL of a stock solution of three internal standards (ILSs) containing 2 µmol/L d_6_-α-13′COOH and d_6_-α-13′OH, respectively, and 46 µmol/L d_6_-α-TOH. A total of 262 µL of the sample mixture were transferred in a 2 mL tube and incubated with 200 µL of a mixture of sulfatase (50 U/mL) and β-glucuronidase (5.100 U/mL) in ammonia bicarbonate (5 mM, pH 6.8) for 2 h at 37 °C. After enzymatic hydrolysis, 1390 µL of acetonitrile (1% formic acid) was added to precipitate the protein, vortexed and centrifuged (5 min, 13,200 rpm, 20 °C). The supernatant was applied directly onto a HybridSPE^®^ column that has been pre-conditioned with acetonitrile and 1% formic acid (FA). The eluted solution was evaporated via SpeedVac at 45 °C, and the residue dissolved in 200 µL 70% methanol (MeOH/H_2_O 70/30 (*v*/*v*), 1 mg/mL AA and 0.1% FA). The quantification (via LC-MS/MS) was achieved using calibration curves of both LCMs from 0.1 to 35 µg/L and 0.1 to 35 mg/L α-TOH prepared in the same solvent.

### 4.5. Liquid Chromatography with Fluorescence Detection

The chromatography (LC-4000-System, FD (FP-1520), JASCO, Pfungstadt, Germany) utilized a Kinetex F5 column (2.1 × 100 mm, 2.6 μm, 100 Å) (Phenomenex, Aschaffenburg, Germany) connected to a SecurityGuard ULTRA cartridge (Phenomenex). The solvent system consisted of methanol/formic acid (1000:1 *v*/*v*, A) and H_2_O/formic acid (1000:1 *v*/*v*, B). The separation was performed with a multi-step gradient scheme as follows: 0 min, 70% B; 3 min, 70% B; 4 min, 80% B; 10 min, 80% B; 12 min, 90% B; 18 min, 90% B; 18.5 min, 90% B (flow rate 0.25 mL/min) and monitored by fluorescence detection at λ_Ex_ = 296 nm and λ_Em_ = 325 nm. The column oven temperature was set to 45 °C.

### 4.6. Liquid Chromatography with Mass Spectroscopy

The LC-MS/MS system consisted of a Dionex UltiMate 3000 UHPLC system equipped with a Kinetex F5 column, coupled to a Bruker AmaZon SL ion trap mass spectrometer with an electrospray ionization (ESI) source (Bruker, Karlsruhe, Germany). For the LC-MS method, a different column format with a smaller particle size of 1.7 µm (2.1 × 100 mm, 1.7 µm, 100 Å) (Phenomenex, Aschaffenburg, Germany) was used. The same LC solvent system was used as described above. Precursor ions were analyzed using base peak monitoring in positive polarity ESI mode (dry gas temperature: 280 °C, flow rate: 8.0 L/min; nebulizer pressure: 2.8 bar; capillary voltage: 4100 V; end plate offset: 500 V) with Bruker Compass Data Analysis software version 4.2. To enhance ionization efficiency, a flow-splitter was employed to reduce the flow rate into the mass spectrometer to 100 µL/min. To further increase sensitivity and improve peak definition, the smartMRM acquisition mode was employed for MS^n^ measurements.

### 4.7. Method Validation

In general, multiple reaction monitoring (MRM) transitions (quantifiers) were used for the acquisition of response linearities to establish the limit of detection (LOD), the limit of quantification (LOQ), and the recovery rate of the metabolites (Table 1). The retention time and the relative ion ratio (qualifiers) were used to identify the metabolites. LOD and LOQ were estimated both by evaluating the signal-to-noise ratio (S/N) of the peaks in the chromatograms of real samples and by calculating from standards curves (instrumental LOD/LOQ) according to Giusepponi et al. [6]. Response linearity was investigated by injecting standard solutions in methanol/water 75/25 *v/v* in an equidistance concentration range of 2.17–54 nmol/L for both long-chain metabolites and 2.32–58 µmol/L for α-TOH. Since no certified reference materials are commercially available, the accuracy and relative recovery, respectively, were determined based on the added labeled standards. The accuracy of the method was calculated from the day-to-day precision data. The mean relative recoveries are given after subtraction of any blank values.

## 5. Conclusions

Research on α-tocopherol and its metabolites has contributed significantly to our understanding of vitamin E metabolism thanks to advances in synthesis, analytical techniques and the use of stable isotope-labeled standards. These developments not only improve the accuracy and precision of metabolic analyses but also open up new avenues for exploring the biological functions, therapeutic potential, and nutritional significance of vitamin E and its metabolites. Further research is needed to elucidate the structures of the yet unidentified metabolites M1, M2, and M3 and to investigate their biological activities and effects on human health. The simultaneous purification and quantification of α-TOH and its metabolites saves work steps so that future studies with a larger number of samples can be carried out in a time- and resource-saving manner. We used MS^2^/MS^3^ transitions for the quantification of α-tocopherol since the MS^2^ signal would lead to saturation at the detector and, thus, limit the calibration range. This principle of mass spectroscopic “dilution” through the use of MS^2^/MS^3^ transitions for α-TOH has shown that, in addition to highly concentrated main vitamins, their metabolites can also be measured at trace levels. The presented method could also be applied to the measurement of vitamin A and its metabolites. Plasma retinol concentrations are in the range of 2–4 µM, and the active metabolite all-trans retinoic acid occurs in 1–3 nM [25]. Future experiments could confirm this new “dilution” approach with deuterated standards.

## Figures and Tables

**Table 1:** Retention times, MRM transitions, limit of detection (LOD), and limit of quantification (LOQ) for α-TOH and its long-chain metabolites in human serum.

Compounds	Retention Time (min)	Ion Species	Qualifier	Quantifier	LOD/LOQ ^a^ nmol/L	Accuracy Recovery (rel.)	Precision RSDr
α-13′-COOH	10.4	[M+H]^+^	461.30	165.00/189.00/414.00	5.3/17.8		
d_6_-α-13′-COOH	10.4	[M+H]^+^	467.30	171.00/194.00/420.00		89%	2.0%
α-13′-OH	11.0	[M+H]^+^	447.30	165.00/429.40	3.4/11.2		
d_6_-α-13′-OH	11.0	[M+H]^+^	453.30	171.00/435.40		94%	6.1%
α-TOH	14.8	[M+H]^+^	431.30 431.30 → 165.00	165.00 137.00	3490/11,635		
d_6_-α-TOH	14.8	[M+H]^+^	437.30 437.30 → 171.00	171.00 143.00		88%	12.6%

^a^ instrumental LOD/LOQ, calculated with calibration data of analyte/ILS area ratios and analyte concentration.

**Table 2:** Recent progress in vitamin E metabolomic studies.

Study	Study Parameters ^a^	Metabolites 13′-COOH [nm/L]	M1 + M2	Σ ^b^	13′-OH [nmol/L]	M3	Σ	TOH [µmol/L]
Bartolini_2021	300 µL plasma endogenous (n = 17)	3.5 ± 1.8	35.1 ± 20.4 + 11.5 ± 6.3	50	2.6 ± 2.1	3.0 ± 0.9	5.6	23.5 ± 3.5
	after supplementation (n = 17)	5.8 ± 1.4	549.2 ± 329.6 + 102.3 ± 76.8	657	18.8 ± 8.7	5.9 ± 2.8	24.7	53.2 ± 10.5
Guisepponi_2019	300 µL plasma endogenous (n = 13)	1.8 ± 1.0	47.8 ± 36.6 + 7.4 ± 7.5	57.0 ± 33.6	3.0 ± 1.6	1.8 ± 1.5	4.8 ± 1.3	31± 6
	NIST plasma (SRM 1950)	1.2 ± 0.4	-	-	3.2 ± 0.5	-	-	16.9 ± 0.2
Guisepponi_2017	500 µL plasma or serum endogenous (n = 2)	1.1–1.3	21–24 + 3.7–4.8	27–29	0.7–1.0	1.1–0.7	1.7–2.8	22.2–23.7
	after supplementation (n = 2)	2.4–2.9	228–295 + 33–43	273–331	4.0–12.0	3.6–10	14–16	38.9–54.2
	endogenous (n = 6)	1.8 ± 0.9	68 ± 40 + 5.3 ± 2.9	75 ± 41				29.6 ± 5.9
Ciffolilli_2015	1000 µL serum endogenous (n = 2)	n.d.	-	-	n.d.			24.2–31.5
	after supplementation (n = 2)	-	-	-	26.5–42.9	-	-	51.6–98.5
this study	700 µL serum endogenous (n = 1) ^c^	-	-	6.7 ± 0.6 (9%) ^d^	-	-	9.2 ± 3.53 (38.4%)	15.7 ± 3.6 (23%)
	after supplementation (n = 1)	-	-	184.5 ± 21.4 (11.6%)	-	-	104.9 ± 47.6 (45.4%)	32.2 ± 10.3 (32%)

^a^ Amount of plasma/serum (µL) for determination of metabolites; endogenous levels of metabolites or after supplementation with α-TOH; number of participants (n). ^b^ Sum of α-13′-COOH + M1 + M2 and α-13′-OH + M3, respectively (Σ). ^c^ Subject of this study is identical to subject 1 in Guisepponi_2017. ^d^ variation coefficient in %.

## Data Availability

The raw data supporting the conclusions of this article will be made available by the authors on request.

## Supplementary Materials

The following supporting information can be downloaded at https://www.mdpi.com/article/10.3390/ph17111405/s1, Figures S1–S3: Evaluation of the purity of d6-metabolites by LC-MS; Table S1: Purity results of synthesized labeled standards.
