# Supplementary material for: A New LC-MS/MS-Based Method for the Simultaneous Detection of α-Tocopherol and Its Long-Chain Metabolites in Plasma Samples Using Stable Isotope Dilution Analysis
Source: Pharmaceuticals (Basel). 2024 Oct 22;17(11):1405. doi: 10.3390/ph17111405 (PMC11597593; doi:10.3390/ph17111405)
Supplement: Supplementary file 1 [file pharmaceuticals-17-01405-s001.zip › pharmaceuticals-3243152-supplementary.pdf]

Figure S1 Evaluation of the Purity of D6-α-Tocopherol by LC-MS

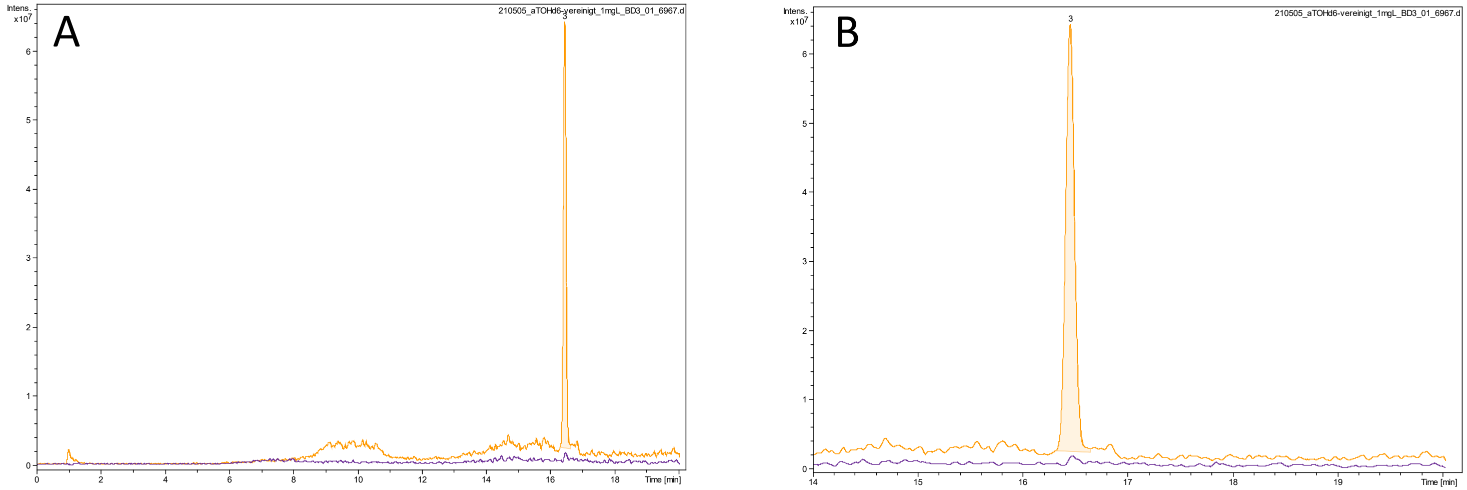

A: Extracted Ion Chromatogram (EIC) showing D6-α-Tocopherol and unlabelled α-Tocopherol. **B** (zoomed section). Extracted Ion Chromatograms (EIC) of D6-α-Tocopherol ($[M+H]^+$, m/z 437.37, orange) and unlabelled α-Tocopherol ($[M+H]^+$, m/z 431.37, purple)

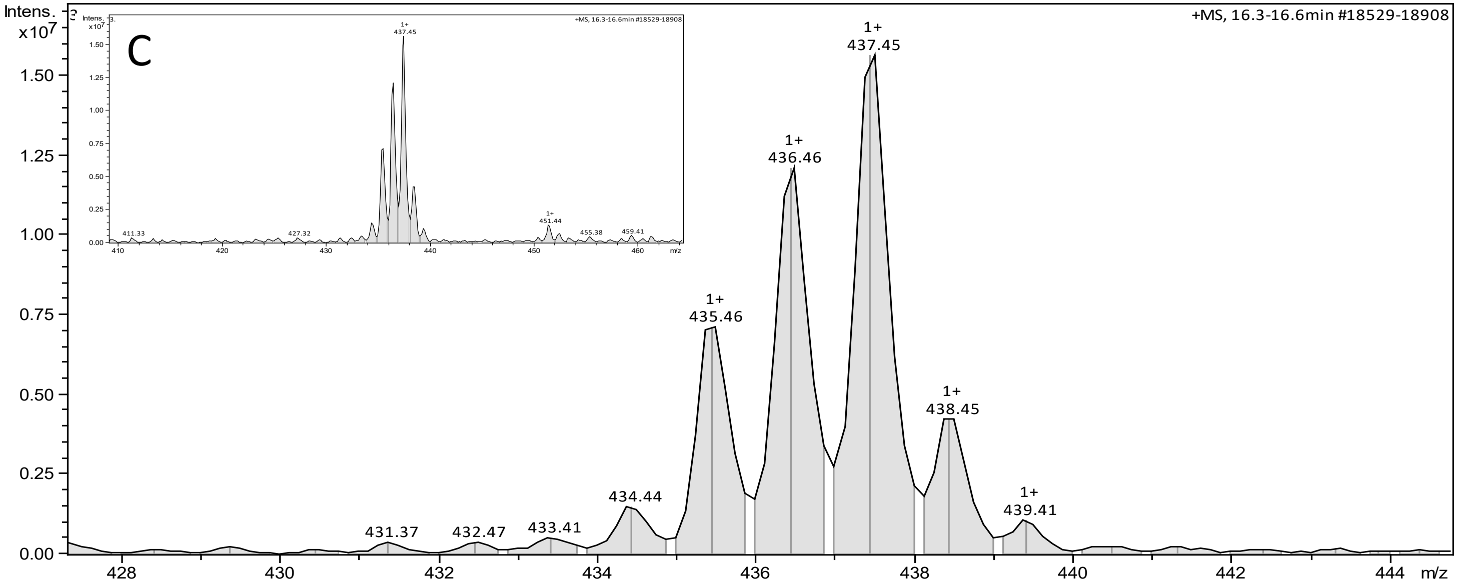

C: Dual Mass Spectrum of D6-α-Tocopherol, with the top left showing the same spectrum over an extended m/z range to display potential adduct ions. The larger spectrum is a highly zoomed-in view aimed at detecting non-labelled α-Tocopherol, which was detected ($[M+H]^+$, m/z 431.37) but is negligible. This confirms the high purity of D6-α-Tocopherol with minimal contamination from non-labelled α-Tocopherol.

Figure S2

Evaluation of the Purity of D6-α-13’-COOH by LC-MS

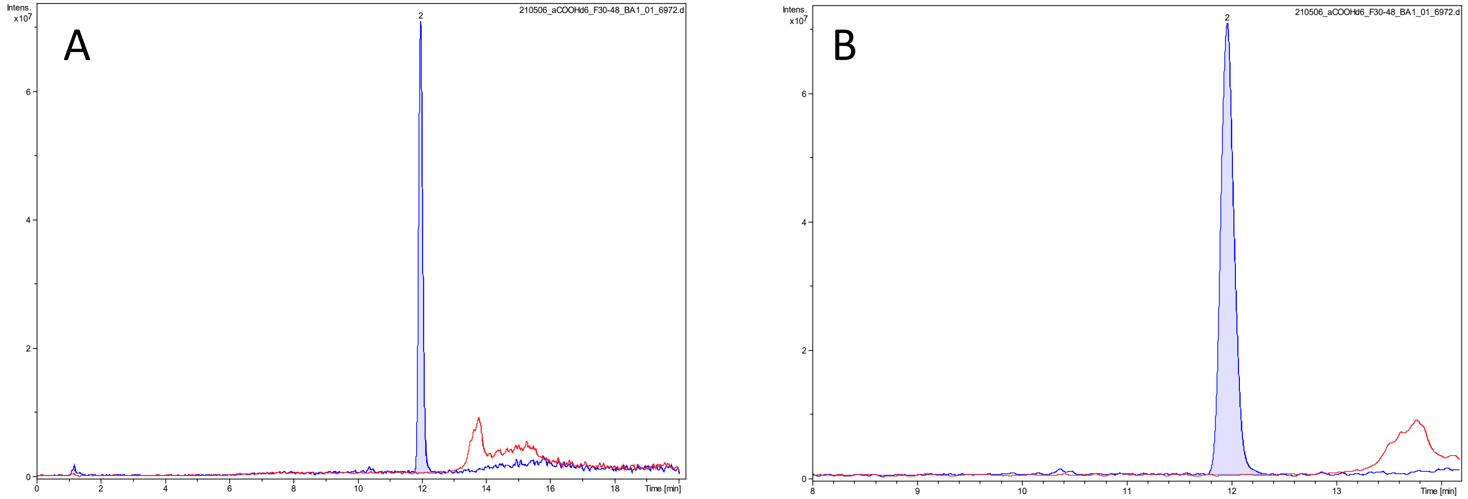

A: Extracted Ion Chromatogram (EIC) showing D6-α-13’-COOH and unlabelled α-13’-COOH. **B** (zoomed section). Extracted Ion Chromatograms (EIC) of D6-α-13‘-COOH ($[M+H]^+$, m/z 467.38, blue) and unlabelled α-13‘-COOH ($[M+H]^+$, m/z 461.37, red)

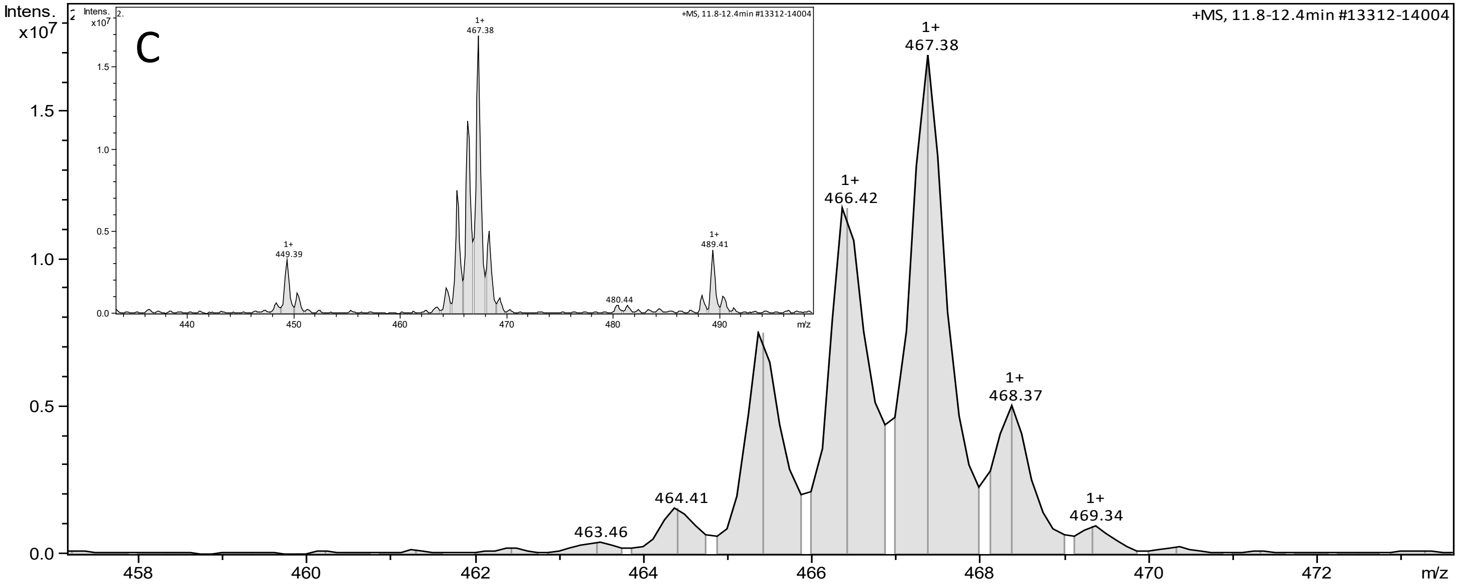

C: Dual Mass Spectrum of D6-α-13’-COOH, with the top left showing the same spectrum over an extended m/z range to display potential adduct ions. The larger spectrum is a highly zoomed-in view aimed at detecting any possible non-labelled α-13’-COOH ($[M+H]^+$, m/z 461.37), which was not observed. This confirms the high purity of D6-α-13’-COOH.

Figure S3 Evaluation of the Purity of D6-α-13’-OH by LC-MS

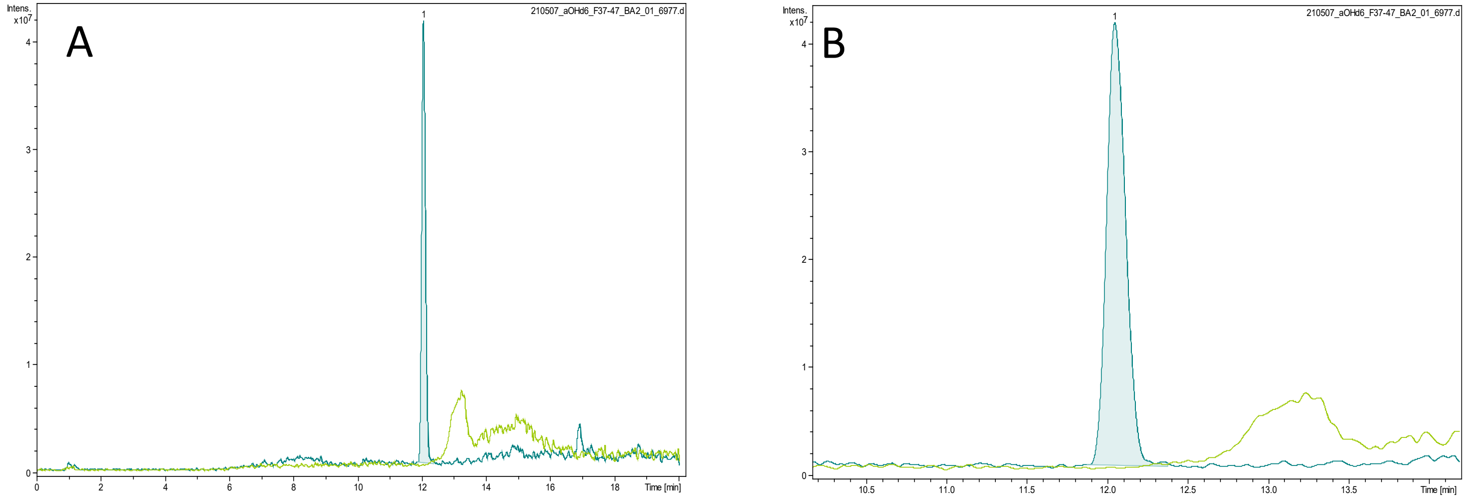

A: Extracted Ion Chromatogram (EIC) showing D6-α-13’-OH and unlabelled α-13’-OH. **B** (zoomed section). Extracted Ion Chromatograms (EIC) of D6-α-13‘-OH ($[M+H]^+$, m/z 453.37, petrol) and unlabelled α-13‘-OH ($[M+H]^+$, m/z 447.37, green)

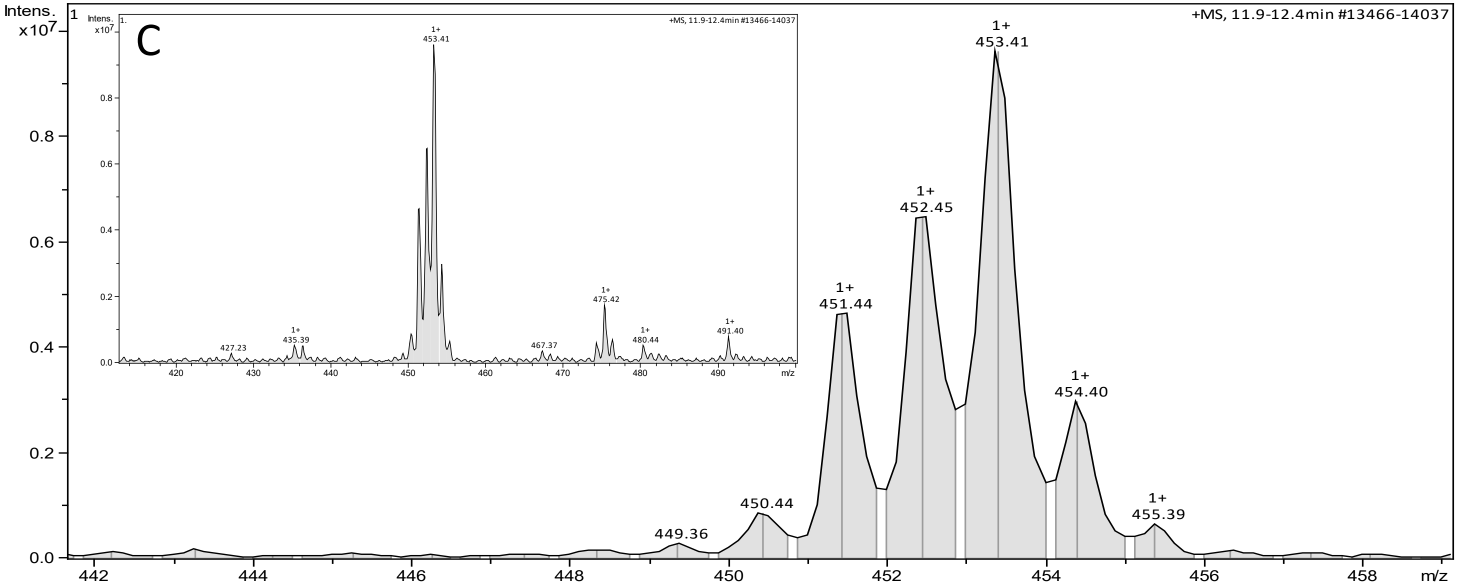

C: Dual Mass Spectrum of D6-α-13’-OH, with the top left showing the same spectrum over an extended m/z range to display potential adduct ions. The larger spectrum is a highly zoomed-in view aimed at detecting any possible non-labelled α-13’-OH ($[M+H]^+$,m/z 447.37), which was not observed. This confirms the high purity of D6-α-13’-OH.

Table S1 Purity Results of synthesized labelled Standards

D6-α-TOH

compound	RT [min]	Chromatogram	Area	Area %
D6-α-TOH	16.8	EIC 467.30 +All MS,	365570784	100.00
α-TOH	16.8	EIC 461.30 +All MS,	3666981	1.00
Purity of D6-α-TOH:		**99.0 %** (impurity with α-TOH 1.00%)		

D6-α-13’-COOH

compound	RT [min]	Chromatogram	Area	Area %
D6-α-13‘-COOH	12.0	EIC 467.30 +All MS,	629785344	100.00
α-13‘-COOH	12.0	EIC 461.30 +All MS,	1383596	0.22
D6-α-13‘-OH	12.8	EIC 453.30 +All MS,	25354578	4.01
α-13‘-OH	12.8		not detectable < S/N	
Purity of D6-α-13‘-COOH:		**95.9 %** (minor impurity of D6-α-13‘-OH 3.8%)		

D6-α-13’-OH

compound	RT [min]	Chromatogram	Area	Area %
D6-α-13‘-OH	12.0	EIC 453.30 +All MS,	629785344	100.00
α-13‘-OH	12.0	EIC 447.30 +All MS,	1383596	0.21
D6-α-13‘- COOH	11.3	EIC 467.30 +All MS,	not detectable < S/N	
α-13‘-COOH	11.3	EIC 461.30 +All MS,	not detectable < S/N	
Purity of D6-α-13‘-OH:		**99.8 %**		
